# Are Wearable ECG Devices Ready for Hospital at Home Application?

**DOI:** 10.3390/s25102982

**Published:** 2025-05-09

**Authors:** Jorge Medina-Avelino, Ricardo Silva-Bustillos, Juan A. Holgado-Terriza

**Affiliations:** 1 Software Engineering Department, Research Centre for Information and Communication Technologies (CITIC-UGR), University of Granada, 18071 Granada, Spain; jorgemedinaa@correo.ugr.es; 2 Faculty of Technology and Innovation, University of Pacifico, Guayaquil 090904, Ecuador; 3 Department of Computing Sciences University of Villanova, Villanova, PA 19085, USA; ricardo.silva@villanova.edu

**Keywords:** artificial intelligence, cardiac health, patient outcomes, remote patient monitoring, wearable devices

## Abstract

The increasing focus on improving care for high-cost patients has highlighted the potential of Hospital at Home (HaH) and remote patient monitoring (RPM) programs to optimize patient outcomes while reducing healthcare costs. This paper examines the role of wearable devices with electrocardiogram (ECG) capabilities for continuous cardiac monitoring, a crucial aspect for the timely detection and management of various cardiac conditions. The functionality of current wearable technology is scrutinized to determine its effectiveness in meeting clinical needs, employing a proposed ABCD guide (accuracy, benefit, compatibility, and data governance) for evaluation. While smartwatches show promise in detecting arrhythmias like atrial fibrillation, their broader diagnostic capabilities, including the potential for monitoring corrected QT (QTc) intervals during pharmacological interventions and approximating multi-lead ECG information for improved myocardial infarction detection, are also explored. Recent advancements in machine learning and deep learning for cardiac health monitoring are highlighted, alongside persistent challenges, particularly concerning signal quality and the need for further validation for widespread adoption in older adults and Hospital at Home settings. Ongoing improvements are necessary to overcome current limitations and fully realize the potential of wearable ECG technology in providing optimal care for high-risk patients.

## 1. Introduction

Health systems, payers, and providers are increasingly focused on finding better ways to deliver care for high-cost patients, those who account for a high proportion of healthcare spending. According to Ref. [1], the average annual expenditure per capita on healthcare for adults with high-needs conditions is nearly three times higher than for adults with multiple chronic diseases, and more than four times higher than the average for all U.S. adults. Rates of hospital use for high-needed adults are more than twice those for adults with multiple chronic conditions. Furthermore, high-needed adults visit the doctor more frequently and utilize more home healthcare.

Hospital at Home (HaH) and remote patient monitoring (RPM) represent innovative healthcare models aimed at enhancing patient care, accessibility, and cost-effectiveness. HaH enables patients to receive acute hospital-level care in the comfort of their homes, while RPM involves continuous monitoring of patients’ health and vital signs from a remote location. Both models leverage advancements in technology and healthcare delivery to revolutionize traditional healthcare practices [1].

Unlike conventional healthcare settings where patients are typically monitored only during brief hospital visits, HaH and RPM offer continuous monitoring, providing a comprehensive view of patients’ cardiac health over time. This continuous surveillance ensures prompt detection and management of potential cardiac issues [2]. However, the question arises: are current wearable devices equipped to deliver the required functionality?

In a seminal review, Bayoumy and colleagues summarize the basic engineering principles of common wearable sensors and discuss their broad applications in cardiovascular disease prevention, diagnosis, and management [3]. They conclude that smart wearable devices have the potential to improve cardiovascular care. However, challenges including device accuracy, privacy concerns, and cost need to be addressed before widespread adoption can occur. The authors propose an ‘ABCD’ guide to help clinicians integrate these devices into their practice. The ABCD guide stands for accuracy, benefit, compatibility, and data governance [3] as follows:

- Accuracy: Clinicians should assess the accuracy of the devices’ hardware sensors and software algorithms. They should look at available literature and regulatory approvals to determine this.
- Benefit: Clinicians should consider the clinical utility of the wearable device and weigh it against its cost. Is there evidence that the device improves patient outcomes?
- Compatibility: Clinicians should assess how the wearable device integrates with their electronic health records and workflow. This includes setting up data review procedures and staff training.
- Data: Clinicians should establish data rights and governance procedures to protect patient privacy. This includes creating data user agreements and privacy policies.

In this paper, Bayoumy et al. [3] analyze how wearable technology can be applied to clinical workflow for optimal cardiovascular patient care employing the ABCD guide as a reference framework. For instance, smartwatches with ECG are able to identify arrhythmias such as atrial fibrillation (AF) [3]. AF is the most prevalent arrhythmia observed in clinical practice, frequently remaining undiagnosed. The ability of certain smartwatches to record an ECG lead I at any time presents opportunities for diagnosing cardiac anomalies [4,5]. AF and flutter are associated with an elevated risk of stroke, heart failure, and other adverse health outcomes [6]. Lifestyle interventions focusing on physical activity, alcohol abstention, and weight loss can reduce the incidence of morbidity. Smart consumer ECG devices could prove particularly beneficial in monitoring prolonged QT intervals, thus preventing the occurrences of arrhythmias with high mortality risks such as ventricular fibrillation [6].

A challenge with smartwatch recordings is their limited detection capabilities, typically restricted to lead I. They often fail to detect ST segment elevations, which may occur in leads II or III. However, some intelligent devices can detect not only three bipolar leads but also unipolar leads AVR, AVL, and AVF. Nevertheless, the 6 leads of the Einthoven triangle have limited reliability in detecting heart attacks, with only approximately 30% to 60% accuracy compared to a gold-standard 12-lead ECG [6,7,8,9].

The market is flooded with inaccurate and inconclusive wearable devices aimed at activity monitoring, such as smartwatches, heart rate monitors, and smart glasses [10,11,12,13]. Recent research urges caution in interpreting metrics from portable devices, highlighting the evolution from motion sensors to advanced photoplethysmogram (PPG) technology for improved precision [2,13,14,15,16,17,18,19]. The availability of portable ECG technology is extensive, which may facilitate the identification of both symptomatic and asymptomatic AF [20,21,22]. Although previous studies have addressed the feasibility of using smart devices to perform manual QT interval measurements, there is a scarcity of data concerning the clinical validation of QT measurements generated by AI from commercially available smart devices [23,24,25,26].

This review seeks to address the following pivotal question: “Are Wearable ECG devices ready for Hospital at Home Applications?” To answer this, we systematically identified and analyzed relevant publications that explore the convergence of wearable technology, the detection and management of cardiac events, and the application of machine learning techniques. These publications were evaluated for their alignment with the key tenets of the ABCD framework—accuracy, benefit, compatibility, and data criteria—required for the seamless and reliable integration of wearable ECG devices into remote patient care workflows.

Some existing reviews have extensively cataloged the types of wearable devices for ECG monitoring, summarized their analytical algorithms [3,6,27], and assessed their accuracy and reliability in various contexts [6], including specific populations like older adults [28], or their integration into current clinical workflows [29,30]; this narrative review adopts a distinct and targeted focus. As highlighted by the scoping review of Zepeda-Echavarria et al. [27], which identified a substantial number of ECG devices (58) but revealed that less than half (26) possess robust clinical evidence for detecting cardiac conditions beyond atrial fibrillation, the landscape is indeed complex and evolving. Their work underscores the heterogeneity of available devices, varying significantly in technical characteristics that directly impact their diagnostic capabilities.

Given the heterogeneity of devices and the varying quality of available studies, a narrative review guided by the ABCD framework allows for a more flexible and comprehensive exploration of the topic. This approach enables us to integrate diverse sources of information, including technical specifications, clinical validations, and user experiences, providing a holistic understanding of the potential and limitations of wearable ECG devices for home use.

To achieve this objective, two key areas are explored in this review based on an analysis of recent research:

- This study investigates the degree to which contemporary wearable devices can offer diagnostic capabilities comparable to the traditional 12-lead ECG, the established gold standard for cardiac diagnosis, with a focus on their potential to use at home. This involves analyzing the accuracy and reliability of wearable ECGs in capturing equivalent information to that of 12-lead ECGs, identifying also any limitations or challenges in achieving this equivalence for home use. Furthermore, it will apply the ABC guideline in non-clinical settings and assess clinical confidence in diagnoses derived from these alternative devices.
- This study also explores the potential of artificial intelligence (AI) to automate the analysis of electrocardiograms obtained from wearable devices. This includes examining various AI algorithms and techniques that can be used to detect arrhythmias and other cardiac conditions more effectively and efficiently. The review also discusses the advantages and challenges of using AI in this context, such as the need for large datasets, the importance of accuracy and reliability, and the potential for personalized medicine.

The rest of the document is organized as follows: Section 2 outlines the methodology of the narrative review; Section 3 explores current wearable ECG technology; Section 4 explores the role of artificial intelligence in the detection of cardiac disease using ECG data; Section 5 examines research focused on both arrhythmias and non-arrhythmias; Section 6 is centered on the detection of acute cardiac episodes; Section 7 explores studies using ECGs for pharmacological interventions; Section 8 discusses the pro/cons of wearable ECG devices; and, finally, Section 9 presents the conclusion, summarizing key findings and outlining future directions.

## 2. Materials and Methods

This study employs a narrative review to investigate whether recent advancements in wearable ECG technology can be effectively utilized for heart disease prevention and control, particularly for Hospital at Home applications. Our analysis is guided by the ABCD framework [3], which highlights key factors for successfully integrating these devices into clinical workflows for optimal cardiovascular patient care.

A narrative review methodology was selected over the more rigid inclusion and exclusion protocols of systematic reviews. This approach allows for a more adaptable and comprehensive synthesis of a heterogeneous body of research, making it particularly appropriate for analyzing the practical applicability of wearable ECGs. It enables a qualitative synthesis of studies that vary in methodology, device type, and clinical implementation strategies.

## 3. ECG Wearable Devices

Wearable medical devices developed to date are intended for use on different parts of the body, including the head, limbs, and torso. These devices serve four primary application domains: health and safety monitoring, chronic disease management, disease diagnosis and treatment, and rehabilitation [29]. However, the wearable medical device sector encounters several significant challenges hindering broader adoption in non-clinical settings. Various key factors influence the acceptance of wearable devices among older adults, health professionals, and caregivers, emphasizing the importance of user-centric design, timely feedback, and affordability. It also underscores the need for integrating behavioral science principles into the development of wearable-based interventions within a comprehensive model of care to improve patient outcomes and satisfaction [31].

Enhancing the comprehension of wearability among both patients and providers could lead to better data quality and aid in the early detection of adverse events like atrial fibrillation. This, in turn, may advance the diagnosis and treatment of cardiovascular diseases, ultimately leading to improved health outcomes [32].

**Figure 1:**
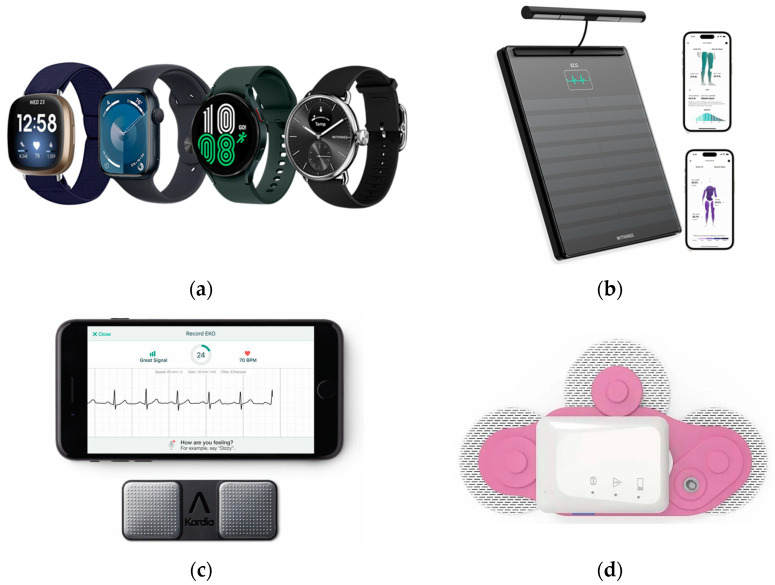
A selection of ECG wearables: (**a**) from left to right, FitBit © Sense, Apple © watch series 4, Samsung © Galaxy Watch 4 and Withings © ScanWatch used in [6]; (**b**) the Withings © body scan is a smart scale that displays ECG data acquired via a multi-electrode grip (6-lead); (**c**) small, portable, medical-grade personal ECG device from AliveCor used in [6]; (**d**) ECG patch for continuous monitoring.

Traditional electrocardiograms (ECGs) are performed in clinical settings using 12 leads to provide a comprehensive view of the heart’s electrical activity. However, portable ECG devices typically employ a reduced number of leads (often one, three, or six) for continuous or on-demand monitoring in non-hospital settings [33]. A diverse array of small, portable, medical-grade ECG devices are readily available to consumers today [34]. ECG technology has been seamlessly integrated into consumer electronics, including multi-sensor sports and fitness trackers, smartwatches, scales, handheld monitors, patches, scales, chest straps, clothing- and shoe-embedded sensors; Figure 1 shows a set of commercially ECG wearable devices used in different studies [5,6]. Specialty consumer ECGs may also come in alternative packaging such as patches or thin, credit card-shaped sensor plates. When the consumer’s device lacks its own mobile SIM card of Wi-Fi, it typically connects to a mobile smartphone.

The heterogeneity of ECG wearables, particularly in reliability, autonomy (battery life), features, and signal quality, necessitates careful consideration for their use in non-clinical environments. The accuracy and clarity of recorded signals are susceptible to factors such as the number of leads, sensor quality, and environmental conditions [35]. Consequently, the adoption of ECG wearables outside of traditional medical settings requires attention to aspects like user operability, the reliability of data transmission in these environments, and the level of clinical confidence in diagnoses derived from these alternative devices.

The sampling rate of the electrocardiogram (ECGs) generated by wearable devices is a crucial factor in the accuracy and detail of the recorded signal. The American Heart Association (AHA) recommends a minimum sampling rate of 500 Hz for standard diagnostic ECGs in adults, but some studies even suggest that a sampling frequency of at least 1000 Hz [36,37] would be desirable for accurate measurements, especially in children. Wearable devices commonly generate sampling rates between 100 and 350 Hz, which is often considered adequate for ECG analysis. This is partly because the ECG is frequently printed for medical professionals on graphical paper with a standard voltage resolution of 10 mm/mV and a time resolution of 25 mm/s for interpretation.

An Important aspect of portable ECG devices Is their ability to analyze the acquired signals. These devices can provide a preliminary diagnosis of cardiac conditions, such as atrial fibrillation, alerting the user to potential problems, but medical attention should be sought. It is important to remember that these analyses are typically for informational purposes and should always be reviewed and confirmed by a qualified healthcare professional.

**Figure 2:**
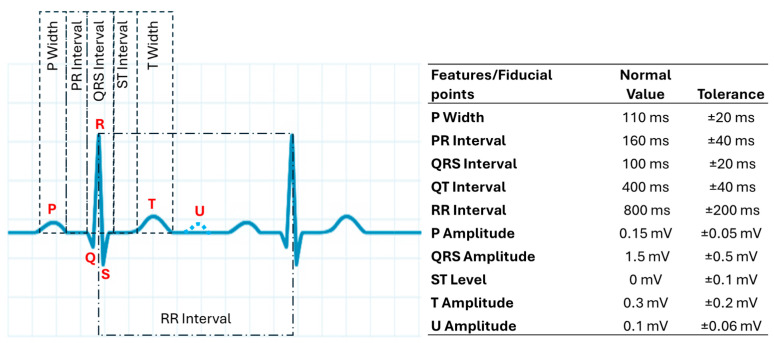
Standard values for amplitudes and intervals, according to the Association for the Advancement of Medical Instrumentation (AAMI), for normal sinus rhythm.

Figure 2 illustrates a typical ECG signal alongside the standard values of key features used in diagnosis. Identifying these features within the ECG is fundamental for signal analysis, especially for automated detection in ECG algorithms. Temporal features are derived from the durations of signal intervals, while morphological features characterize the shape of specific segments, like the ST segment or the QRS complex. By examining the amplitudes and intervals and their precise fiducial points, the function of the heart’s electrical conduction system can be evaluated [38].

## 4. Artificial Intelligence for Analyzing ECG

Artificial intelligence (AI) is increasingly utilized in medicine, integrating para-clinical exams with clinical findings to enhance diagnostic accuracy and facilitate timely interventions. AI-driven ECG analysis has proven instrumental in identifying cardiac anomalies, predicting atrial fibrillation [39], and assessing ejection fraction. These advancements hold significant promise for home-based monitoring, where AI-enabled wearable ECG devices can provide continuous cardiac assessment and early anomaly detection, improving patient outcomes in remote settings.

The application particularly of machine learning and deep learning holds significant potential for improving cardiac diagnosis, being able to identify both ischemic events and a wide range of arrhythmias by examining distinct sets of ECG characteristics [40]. This comprehensive assessment assists clinicians by covering both structural/ischemic issues and rhythmic disturbances in cardiac electrical activity.

Specifically, AI is instrumental in detecting ST-elevation myocardial infarction (STEMI) and non-ST-elevation myocardial infarction (NSTEMI). STEMI, caused by complete coronary artery blockage, is characterized by a distinctive ST elevation, a key indicator of acute myocardial injury [41]. Detecting these conditions involves analyzing specific patterns of ST segment changes (including elevation and depression), as well as alterations in T waves and the potential development of pathological Q waves over time, all of which contribute to the comprehensive diagnosis of myocardial infarction. NSTEMI, often resulting from oxygen supply–demand imbalances, typically lacks the prominent ST elevation but is also diagnosed through the analysis of these evolving ECG features.

Furthermore, AI plays a crucial role in arrhythmia detection, which focuses on identifying irregularities in the heart’s rhythm. This analysis involves examining the timing and regularity of heartbeats, the origin of the electrical impulse, and the heart’s conduction pathways. Key features analyzed include the intervals between heartbeats (RR, PR, and QT intervals), the presence of extra or missed beats, and the morphology of the P wave and QRS complex to determine the origin and type of the abnormal rhythm.

In fact, Ref. [39] presents a solution that utilizes the IoMT to collect physiological data from wearable and other connected medical devices, such as continuous heart rate and ECG monitoring, along with blood pressure. In this case, advanced AI algorithms TabNet and catBoost are employed to improve data processing efficiency, feature selection, and predictive accuracy. The model utilizes a dataset that includes diverse patient profiles and risk factors, allowing it to adapt to different demographic and clinical scenarios.

## 5. Wearable Devices for Arrhythmic and Non-Arrhythmic Diseases

The advent of wearable electrocardiogram (ECG) devices, coupled with the power of machine learning and deep learning algorithms, is revolutionizing cardiac diagnostics. By continuously monitoring the heart’s electrical activity in everyday settings, these AI-driven wearable systems offer unprecedented opportunities for early diagnosis, personalized risk assessment, and improved management of a wide range of cardiovascular conditions, extending beyond traditional clinical ECG assessments.

Rjoob et al. [42] analyzed machine learning (ML) applications in ECG interpretation, highlighting their relevance for homecare. Their review of 757 studies indicated that the majority (400/757) focused on classifying cardiac anomalies, with a strong emphasis on arrhythmia (AR) (202/400) and non-arrhythmic (Non-AR) conditions (62/400). Atrial fibrillation detection was the most studied arrhythmic condition (57.53%), followed by premature ventricular contractions and ventricular fibrillation. In the non-arrhythmic category, ischemia and infarction detection accounted for 44.79% of studies. The ability of AI to detect these conditions in real time through wearable ECG devices is crucial for early intervention in home-based settings.

Salama et al. [26] demonstrated the use of AI with the Physionet MIT-BIH Arrhythmia dataset, applying preprocessing techniques such as data truncation, augmentation, and feature extraction. They employed ML algorithms, including k-nearest neighbors (KNN), Random Forest (RF), Support Vector Machines (SVM), Decision Trees (DT), and Deep Learning Convolutional Networks (CNN). Their evaluation metrics, presented in Table 1, show that CNN-based approaches yielded superior accuracy. This underscores the feasibility of integrating CNN models into wearable ECG systems to enhance real-time arrhythmia detection at home.

Sanjay et al. [43] developed an 11-layer CNN model based on VGGNet to classify ECG images into eight arrhythmic categories. Using R-peak segmentation and k-fold cross-validation, they optimized accuracy across conditions such as premature atrial contractions (APC), left bundle branch block (LBBB), and normal sinus rhythm. Such deep learning architectures can be adapted to home-based ECG systems to enhance diagnostic precision and reduce false alarms in continuous monitoring. The precision, recall, and F1-Score metrics for each category are presented, along with overall accuracy, macro average, and weighted average in Table 2. The arrhythmic diseases with the highest percentage of prediction in the three metrics considered were VEB, LBBB, and PAB, all above 99%.

**Figure 3:**
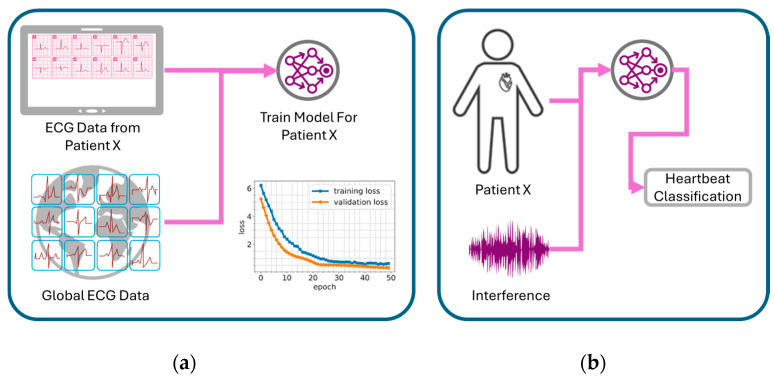
(**a**) Patient-specific training. (**b**) Continuous ECG monitoring and heartbeat classification in real time. Adapted from Ref. [44].

**Figure 4:**
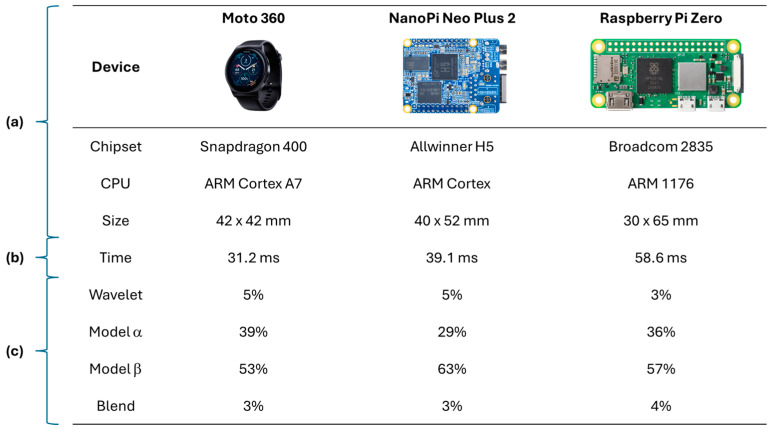
(**a**) Hardware platforms. (**b**) Measured execution time. (**c**) Distribution of the execution time. Adapted from Ref. [44].

Saadatnejad et al. [44] proposed a personalized AI approach for real-time arrhythmia monitoring. Their approach leverages both patient-specific (local) ECG data and a broader (global) ECG dataset containing common characteristics of the studied arrhythmias (as illustrated in Figure 3) to create tailored classification models. Their research also assessed the viability of implementing AI-driven ECG analysis on wearable devices, evaluating hardware such as Moto 360 smartwatch (Motorola Mobility LLC, Chicago, IL, USA), Nano Pi Neo Plus 2 (FriendlyElec, Shenzhen, Guangdong, China), and Raspberry Pi Zero (Raspberry Pi Foundation, Cambridge, Cambridgeshire, UK) (depicted in Figure 4). This patient-centric strategy holds significant promise for homecare settings, where continuous, adaptive monitoring is essential for personalized cardiac health management.

Then, in general, the development of health systems employs high performance embedded devices (e.g., raspberry pi in [44]), where accuracy and specificity metrics can be obtained with high percentages of prediction in the classification of arrhythmias, with the benefit of early detection in the prevention of heart disease and follow-up of already diagnosed patients. In addition, the use of this hardware guarantees the compatibility of the solutions to be highly recognized equipment in intelligent solutions that allow managing patient data in an interoperable manner.

The increasing availability of data from connected health devices enables the application of machine learning (e.g., SVC, Random Forest, XGBoost, LinearSVC) and deep learning algorithms for automated ECG classification in arrhythmia studies. For instance, Tang et al. [45] demonstrate significant improvements in accuracy, with XGBoost optimized by the JADE algorithm achieving an F1-score of 0.8742. The potential benefit lies in enhancing the diagnosis and monitoring of heart conditions by providing reliable automated ECG interpretation for physicians. The focus on ECG data, including that from wearable devices, suggests compatibility with existing and emerging cardiac healthcare technologies. While the study highlights the use of substantial data volumes, with PCA applied for dimensionality reduction, specific details regarding dataset diversity and characteristics are not elaborated in these excerpts. Notably, the analysis does not explicitly address fairness considerations concerning potential biases in the data or the generalizability of the models across diverse patient populations.

Commentary by Rahman et al. [46] in Mayo Clinic Proceedings: Digital Health evaluates the application of federated learning in cardiology through the ABCD lens. Regarding accuracy, the paper focuses on the potential for enhanced CVD detection and personalized risk stratification through collaborative model training on diverse, decentralized data, without providing specific current performance metrics. The benefit lies in creating more robust and privacy-preserving AI models for cardiology, leveraging varied patient data from multiple sources and enabling continuous model refinement. Compatibility is addressed by positioning federated learning as a solution for analyzing IoT data in cardiology, integrating with existing and emerging data streams while mitigating privacy concerns. The paper acknowledges challenges related to fairness, specifically the “non-IID” nature of heterogeneous patient data across different stakeholders, which could impact model generalizability. Finally, concerning data, federated learning’s core strength is its ability to learn from distributed “vast IoT datasets” without centralizing sensitive information, although the authors also notes potential issues with data heterogeneity and signal quality across different sources.

**Figure 5:**
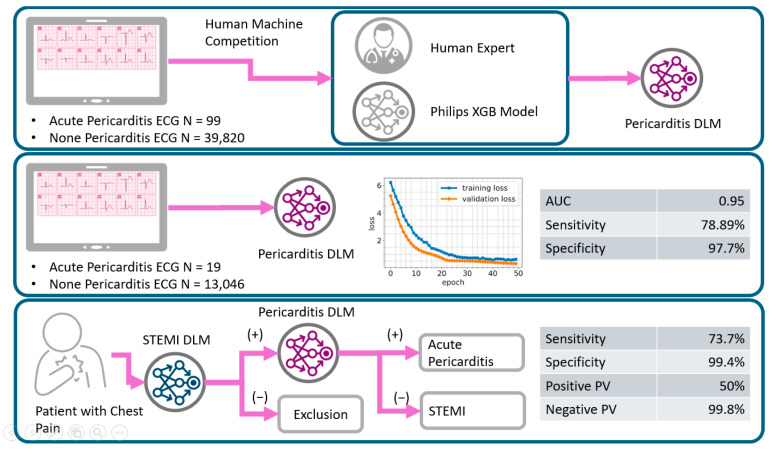
Flow chart detailing the development, validation, and potential future applications of the pericarditis deep learning model (DLM). Adapted from Ref. [47].

Yu-Lan et al. [47] explored the integration of AI-enhanced feature extraction methods for ECG classification in mobile and wearable devices. Their study emphasized the importance of optimizing computational efficiency while maintaining diagnostic accuracy, a key consideration for home-based applications. They proposed a hybrid deep learning framework that effectively balances power consumption with real-time processing, enabling accurate arrhythmia detection in resource-limited environments. Their findings support the ongoing evolution of AI-ECG systems tailored for home monitoring, highlighting the need for robust yet energy-efficient algorithms, as seen in Figure 5.

## 6. Wearables for Acute Cardiac Events

**Figure 6:**
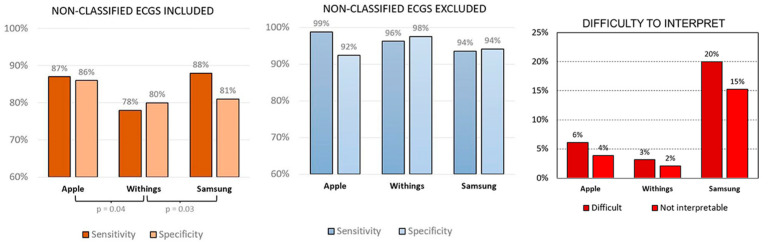
Automatic diagnosis of AF for the Apple Watch Series 5, Samsung Galaxy Watch Active 3, and Withings Move ECG (*p* = 0.02 between Withings and Apple) and (*p* = 0.03 between Samsung and Withings) [2].

Recent guidelines recognize the potential value of capturing ECG signals using smartwatches for diagnosing atrial fibrillation [31,32]. In terms of accuracy, comparative studies of atrial fibrillation diagnostic algorithms, such as the one conducted by Abu-Alrub [2], evaluate detection using wearable devices such as the Apple Watch Series 5, Samsung Galaxy Watch Active 3, and Withings Move ECG through a prospective, non-randomized, and blinded clinical trial. The trial involved 100 consecutive patients in RS sinus rhythm and 100 consecutive patients with persistent or permanent AF, excluding those with atrial flutter, permanent pacemakers, or implantable automatic defibrillators. All patients underwent a 12-lead ECG, serving as the reference standard. Sensitivity, specificity, positive predictive values, and negative predictive values for smartwatch ECGs were calculated for each of the three smartwatch models. Analysis of variance (ANOVA) tests were used to compare the three groups. All automated smartwatch algorithms demonstrated high sensitivity and specificity for diagnosing AF (benefit for early detection and management); however, the Withings smartwatch exhibited lower sensitivity and specificity compared to the Apple and Samsung, as shown in Figure 6. The compatibility of these devices for personal use is high, and the data generated allow for remote monitoring and potential sharing with clinicians.

**Figure 7:**
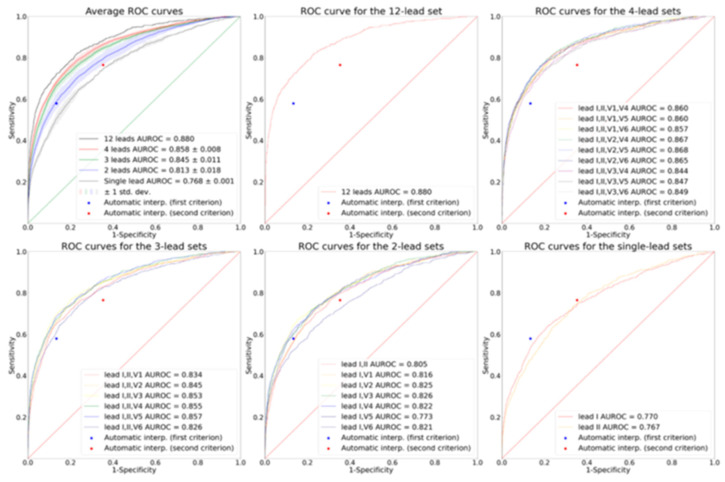
Model performance as the number of leads increases. Graph was obtained by using results from the paper (12-lead sets: AUROC 0.880; 4-lead sets: AUROC 0.858, SD 0.008; 3-lead sets: AUROC 0.845, SD 0.011; 2-lead sets: AUROC 0.813, SD 0.018; single-lead sets: AUROC 0.768, SD 0.001) [35].

Although atrial fibrillation detection is well established in smartwatches, analyzing the heart’s electrical activity from various spatial locations using multiple leads has been considered essential for accurately detecting cardiac disorders such as myocardial infarction, pulmonary embolism, and acute left or right heart failure [34,35]. Therefore, the standard 12-lead ECG remains the most widely used evaluation tool among doctors for assessing heart health. Studies have shown that while individual ECG signals are asynchronous, they become synchronous when grouped together [35]. Regarding benefit, the ability to derive sets of asynchronous ECG leads from smartwatch recordings to mimic sequentially recorded ECG leads offers the potential to broaden diagnostic capabilities beyond single-lead limitations. For instance, a 4-lead subset consisting of leads I, aVR, V1, and V4 from the ECG report is fully asynchronous. The study presents receiver operating characteristic (ROC) curves for various target lead combinations, indicating that increased lead information improves diagnostic accuracy for conditions beyond arrhythmias. While the compatibility of obtaining multiple leads with current smartwatches is limited by their asynchronous nature, the findings suggest that multiple AI-based ECG algorithms can be implemented on these devices. Such implementation can enable timely diagnosis, broaden accessibility to results, and potentially reduce mortality among populations with cardiovascular diseases outside the hospital setting (benefit). As shown in Figure 7, model performance generally improves as the number of leads increases; therefore, in emergency situations, the authors recommend measuring at least three leads (i.e., I, II, and V5) and ideally more than four leads (i.e., I, II, V2, and V5) to minimize the risk of missing acute myocardial infarction. The data generated from these asynchronous multi-lead approaches require sophisticated AI interpretation.

Strik et al. [48] explored alternative recording positions for smartwatches and discovered that recording with only a single tracing achieved improved accuracy in detecting ST/T wave abnormalities when the smartwatch was positioned on the left ankle or on the chest wall (positions V1 and V6). This alternative positioning yielded a sensitivity of 77% and a specificity of 92%, with a *p* value < 0.01 compared to standard wrist recording [49,50]. This enhanced accuracy through modified usage (compatibility) could offer a potential benefit for early identification of conditions like ischemia, even with a single-lead device. The data interpretation still relies on algorithms to identify these subtle ST/T wave changes.

**Figure 8:**
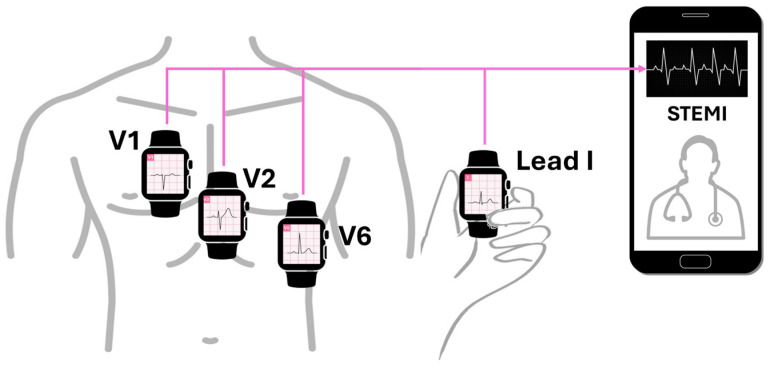
ECG Smartwatch of a patient with symptoms of chest pain and 12-lead ECG and confirmed previous STEMI. Typical use location on left wrist records a normal ECG of Einthoven lead I. Adapted from Ref. [48].

In another study involving 100 patients (54 with STEMI, 27 with NSTEMI, and 19 healthy individuals), recording multiple smartwatch ECG tracings (leads 1 to 3, V1–V6) demonstrated correspondence with the standard ECG for identifying normal patients, those with ST segment elevation changes, and those with ST segment elevations not related to myocardial infarction [4]. These findings indicate that multi-lead smartwatch tracings align with traditional ECG waveforms observed in acute coronary syndromes, supporting the potential ability to adequately detect ST-segment elevation and provide accurate diagnoses as shown in Figure 8.

**Figure 9:**
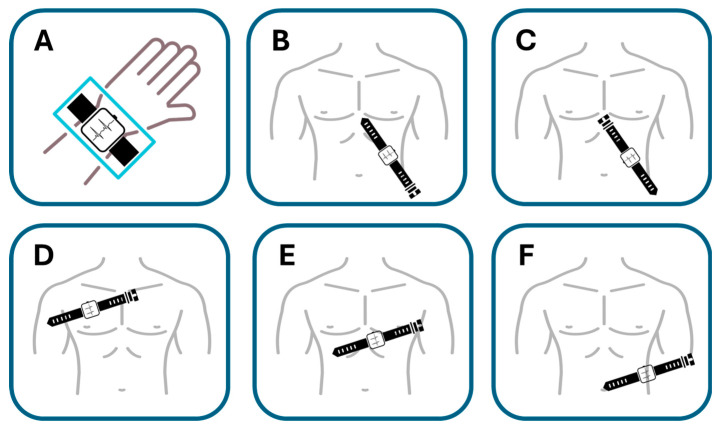
Placement of smartwatch for recording Einthoven and precordial leads. (**A**) Einthoven lead I. Recording between the left wrist and the right index finger. (**B**) Einthoven lead II. Recording between the left abdominal region and the right index finger. (**C**) Einthoven lead III. Recording between the left abdominal region and the left index finger. (**D**) Right Wilson lead (Wr). Recording at the right fourth parasternal intercostal space. (**E**) Medial Wilson lead (Wm). Recording from the midclavicular line of the fifth intercostal space. (**F**) Left Wilson lead (Wl). Recording from the fifth intercostal space of the left midaxillary line. Adapted from Ref. [51].

Samol et al. [50]. utilized an Apple Watch Series 4 for the six single-lead ECG recordings immediately following the acquisition of a 12-lead ECG. The procedure for recording Einthoven leads I to III with the Apple Watch followed the previously described protocol [34]. In summary, Einthoven I was recorded with the Apple Watch on the left wrist and the right index finger on the crown (Figure 9A), Einthoven II was recorded with the watch on the left lower abdomen and the right index finger on the crown (Figure 9B), and Einthoven III was recorded with the watch on the left lower abdomen and the left index finger on the crown of the head (Figure 9C). Pseudounipolar Wilson-type thoracic leads corresponding to the locations of V1, V4, and V6 on the standard 12-lead ECG were recorded. The right Wilson type (Wr) (Figure 9D) corresponded to V1 and was recorded with the smartwatch placed in the right fourth parasternal intercostal space.

The medial Wilson type (Wm) (Figure 9E) corresponded to V4 with the smartwatch placed in the fifth intercostal space in the midclavicular line. The left Wilson type (Wl) corresponded to V6 with the smartwatch in the fifth intercostal space in the left mid-axillary line (Figure 9F). For all Wilson-type chest lead recordings, the smartwatch was placed on the three described locations on the chest, the right index finger was placed on the crown, and the left hand surrounded the right wrist.

**Figure 10:**
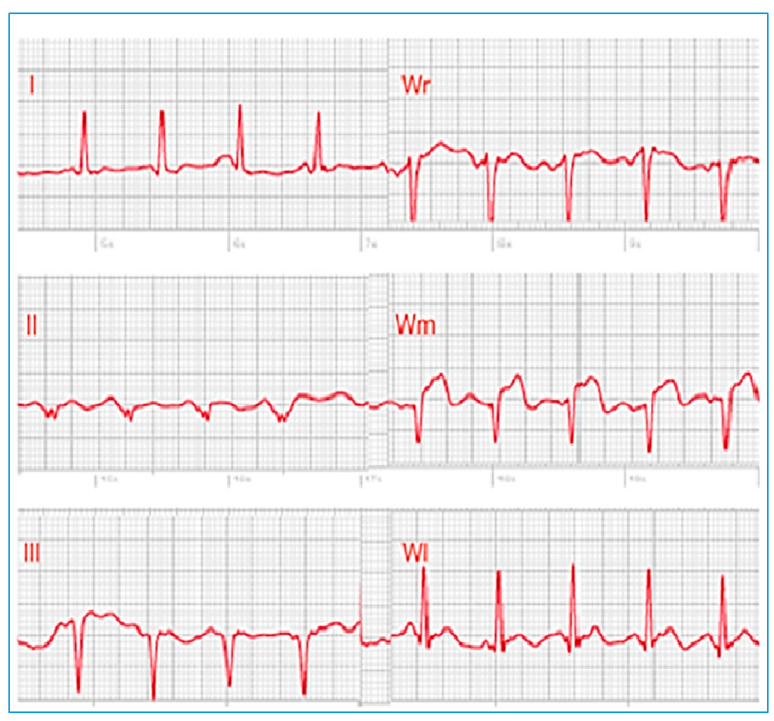
Electrocardiogram with ST segment elevation in Wm (V4). (Wr V1 and WL V6). Adapted from Ref. [50].

This study explored the compatibility of using a single-sensor device in a non-standard manner to gather more comprehensive cardiac data. The significant finding was that in two male patients with acute anterior myocardial infarction, ST segment elevations—a key indicator of MI—could be clearly recognized in the smartwatch thoracic lead recordings and correctly correlated with the presumed occluded vessel (accuracy for critical condition detection, albeit in a limited sample). While the sequential nature of the recordings presents challenges for real-time emergency use (compatibility limitations), it suggests a potential benefit for enhanced diagnostic information beyond a standard single-lead recording in certain scenarios. Figure 10 shows the ECG detecting ST segment elevation.

The study by Choi et al. [51] investigated the use of smartwatch ECG and artificial intelligence (AI) in detecting acute coronary syndrome (ACS) compared to the traditional 12-lead ECG presents promising findings for the application of wearable technology in advanced cardiac diagnostics. In terms of accuracy, the research indicates that the AI-enhanced smartwatch ECG demonstrated a level of sensitivity and specificity that warrants further exploration as a potential screening tool for ACS. The capability of identifying ECG changes beyond basic arrhythmias, which could be indicative of ACS, represents a significant advancement in the diagnostic potential of wearable devices. This improved accuracy translates to a notable benefit for Hospital at Home (HaH) applications. The potential for early detection of ACS in a home setting, utilizing a readily accessible device like a smartwatch, could lead to a crucial reduction in the time to diagnosis and subsequent medical intervention, ultimately improving patient outcomes and lowering morbidity and mortality rates. Furthermore, the possibility of continuous or intermittent monitoring could aid in the identification of atypical ACS presentations. Regarding compatibility, smartwatches offer inherent advantages in terms of personal usability and the feasibility of continuous data acquisition. The integration of AI algorithms, whether directly on the device or via a linked platform, enhances the analytical capabilities of the smartwatch ECG. However, successful implementation in HaH settings necessitates careful consideration of compatibility with existing clinical workflows, the requirement for physician oversight in interpreting AI-flagged events, and the establishment of seamless data transfer mechanisms to hospital information systems. Finally, the utilization of AI-analyzed ECG data obtained from a personal wearable device for the potential diagnosis of a critical condition like ACS raises significant data governance concerns. Ensuring the robust security and privacy of this sensitive health information, along with adhering to the relevant regulatory frameworks governing AI-driven medical devices, is paramount for the ethical and responsible integration of this technology within HaH environments.

The review article by Bayoumy et al. [3] provides a comprehensive overview of smart wearable devices in cardiovascular care and offers insights into their current state and future directions. In terms of accuracy, the paper discusses the varying levels of accuracy achieved by different wearable devices for various cardiovascular parameters, including heart rate monitoring, arrhythmia detection (particularly atrial fibrillation), and even the emerging capabilities for blood pressure and oxygen saturation monitoring. It highlights the importance of validation studies and the need for continuous improvement in sensor technology and algorithms to ensure clinical-grade accuracy. Regarding benefit, the review emphasizes the significant potential benefits of smart wearables in cardiovascular care, including continuous and remote patient monitoring, early detection of cardiovascular events, improved patient engagement in their health, and the facilitation of personalized medicine approaches. These benefits are particularly relevant for Hospital at Home applications, enabling proactive management and potentially reducing the need for frequent hospital visits. The compatibility of smart wearable devices with existing healthcare ecosystems is also a key focus of the article. It discusses the challenges and opportunities related to data integration with electronic health records (EHRs), interoperability between different devices and platforms, and the need for user-friendly interfaces for both patients and clinicians to ensure seamless adoption in clinical practice and HaH settings. Finally, the paper addresses crucial aspects of data governance, including data privacy, security, regulatory considerations, and the ethical implications of collecting and utilizing large volumes of personal health data from wearable devices. Establishing robust frameworks for data management and ensuring patient trust are identified as critical factors for the widespread and responsible implementation of these technologies in cardiovascular care and within HaH programs.

## 7. Smart Watches for Pharmacological Interventions

The focus on studying arrhythmias remains highly valuable for both the scientific community and society at large, especially considering health emergencies like the COVID-19 pandemic caused by the severe acute respiratory syndrome coronavirus 2 (SARS-CoV-2), which affected over 11 million people and resulted in more than half a million deaths [52]. In response to this crisis, exploring pharmacological interventions became imperative, with antiviral medications being one such intervention. However, the potential for cardiac toxicity, particularly in relation to severe proarrhythmia, is a major concern with many proposed treatments, including lopinavir/ritonavir, chloroquine/hydroxychloroquine (HCQ), and azithromycin (AZM) [30,53]. Monitoring the QT interval and heart rate is crucial for ensuring the safety of these medications [53].

**Figure 11:**
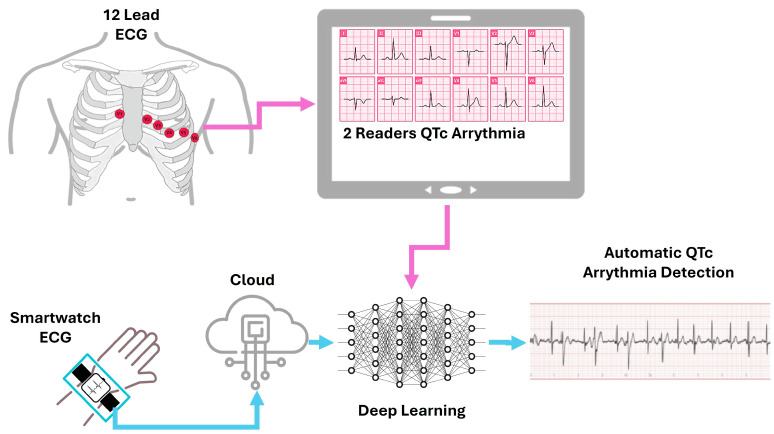
QTc interval assessed by AI on single-lead smartwatch ECG (AI-QTc) with QTc measured by conventional 12-lead ECG in patients with early-stage COVID-19 treated with HCQ-AZM. Adapted from Ref. [52].

While single-lead ECG devices coupled with artificial intelligence (AI) have been endorsed by the US Food and Drug Administration for detecting atrial fibrillation, their feasibility and diagnostic accuracy for evaluating parameters like QTc interval and QTc measurement with AI remain less explored [52]. The Cardiologs Platform, powered by a deep neural network algorithm, offers a cloud-based solution for ECG interpretation. By integrating with devices like the Withings Move ECG (compatibility with existing platforms), this platform enables self-recorded ECGs to be analyzed directly by AI, as seen in Figure 11 [52]. The study found good agreement between manually measured QTc duration on standard 12-lead ECG and AI-assessed QTc on single-lead smartwatch recordings (accuracy in measuring a critical parameter), even across various points during the study. This suggests a potential benefit for remote monitoring of drug-induced QTc prolongation, crucial for ensuring medication safety. Importantly, in this young population with early-stage COVID-19 and mild to moderate symptoms, no significant QTc prolongation or life-threatening arrhythmias were detected, providing initial data on the feasibility in this specific context.

Sarma et al. [54] highlight the potential of ML to improve the interpretation of diagnostic tests like ECGs and echocardiograms and to enhance risk stratification and prognostication. However, they do not explicitly quantify the current accuracy levels achieved, suggesting this is an area of ongoing development and validation. The anticipated benefits are significant, including optimized triaging and discharge, discovery of disease sub-phenotypes for conditions like cardiogenic shock and heart failure, enhanced routine clinical care, facilitated medical education, and the potential for individualized therapies. While the document does not detail specific compatibility with existing infrastructure, the focus on leveraging current diagnostic tests (ECG, echocardiograms) suggests an effort to integrate with established workflows. However, the need for “improved clinician understanding of AI” points to a potential challenge in terms of user compatibility and adoption. The success of ML relies heavily on data. The document implies the availability of diagnostic test data and patient outcome data for training and validation. However, it does not specify the volume, quality, or accessibility of these data, which are crucial for robust and reliable ML applications in cardiac care.

Predel et al.’s [55] ethical analysis of smartwatch-based atrial fibrillation screening, utilizing Beauchamp and Childress’s principles, reveals significant concerns across the ABCFD framework. The accuracy is questionable due to a lack of evidence showing improved outcomes and a high rate of false positives. While the benefit of early detection for stroke prevention exists and patient proactivity could enhance autonomy and beneficence with proper guidance, the current implementation raises issues. Compatibility with consumer use is high, but ethical compatibility with the doctor–patient relationship is threatened by potential over-reliance and insufficient education. Fairness is a major concern, with risks of socioeconomic disparities in access and potential discrimination against underrepresented ethnic minorities in training data. Finally, the data privacy and security of sensitive medical information held by private companies present considerable ethical and legal challenges, leading the authors to conclude that current smartwatch-based atrial fibrillation detection is ethically problematic.

## 8. Discussion

Although artificial intelligence in electrocardiography (AI-ECG) has traditionally focused on standard 12-lead hospital-grade ECGs, current analyzed research reveals that single-lead and multi-lead wearable ECGs, enhanced by deep learning, can achieve comparable diagnostic accuracy.

Nevertheless, Ardeti et al. [38] highlight that the accuracy of these AI-driven systems heavily relies on the quality of the input data, underscoring the “garbage in, garbage out” principle. To ensure data quality, they propose a five-stage process for improved ECG arrhythmia detection. This five-stage process is composed by (1) data acquisition, (2) preprocessing, (3) feature engineering, (4) feature optimization, and (5) feature classification. Among these, preprocessing plays the most critical role, as raw ECG signals are often distorted by noise and artifacts that can compromise AI model performance. Then, the primary objective of preprocessing is to enhance signal clarity by reducing unwanted distortions. Common noise sources affecting smart ECG devices include the following:

- Baseline wander: Low-frequency fluctuations (0.15–0.3 Hz) due to electrode–skin impedance shifts, respiration, and body movements.
- Electrosurgical noise: High-frequency interference (100 kHz–1 MHz) from nearby electronic equipment.
- Electrode contact noise: Signal disruptions caused by poor skin–electrode contact.
- Muscle noise: Electrical activity from non-cardiac muscle contractions.
- Motion artifacts: Impedance variations at the skin–electrode interface resulting from physical movement, leading to ECG signal shifts.

These factors, which can lead to data distortion (as further detailed in publications summarized in Table 3), present significant challenges for achieving the high levels of accuracy required for reliable “Hospital at Home” applications, and ultimately limit their benefit in clinical settings.

Table 3 presents a summary of the limitations, advantages, and disadvantages identified in the research studies selected for this work. Specifically, this table offers a concise overview of the pros and cons associated with the use of ECG wearable devices, which is essential information for evaluating their feasibility and potential home application in non-clinical settings.

Furthermore, the practical benefit of many current wearable devices is limited by their lack of comprehensive analytical capabilities. This necessitates review by trained clinicians before the data can be actionable, impacting on the efficiency and scalability needed for widespread HaH implementation. As outlined in publications detailed in Table 4, the current limitations in accuracy (sensitivity and specificity), the restricted number of leads, and the variability in electrode placement across different wearable devices hinder their ability to provide diagnostic information comparable to standard ECGs for a wide range of cardiac conditions. This directly impacts their readiness for use in more complex HaH scenarios beyond basic arrhythmia detection.

Table 5 categorizes key references according to the ABCD (accuracy, benefit, compatibility, and data) classification, offering a structured assessment of smartwatch-based ECG analysis. As evident in this categorization, accuracy emerges as a primary focus, evaluating the reliability of ECG measurements, atrial fibrillation (AF) detection, and multi-lead ECG interpretations. The potential benefit of smartwatch ECG technology is also well documented, particularly in terms of clinical relevance, early disease detection, and improved accessibility for remote patient monitoring. Compatibility, which pertains to the integration of smartwatch ECG systems into clinical workflows and their ease of use for patients (especially older adults), is addressed in some studies but remains an area requiring further exploration to ensure seamless adoption in HaH programs. Finally, data governance, including privacy and security, is a crucial consideration for the widespread implementation of wearable ECGs in remote settings and needs robust frameworks to ensure patient trust and regulatory compliance.

## 9. Conclusions

The successful integration of wearable ECG devices into healthcare, particularly for “Hospital at Home” applications, fundamentally relies on the development of systems capable of consistently acquiring high-quality ECG signals and employing AI-ECG models that deliver accurate and reliable diagnostic results. As highlighted throughout this review, while current wearable technology has demonstrated significant progress in diagnosing specific conditions like atrial fibrillation with acceptable accuracy and potential benefit for early detection, several limitations hinder their widespread adoption for comprehensive cardiac monitoring in remote settings. The challenges associated with noise and artifacts in the acquired signals directly impact the reliability required for clinical decision making.

The application of the ABCD framework underscores the current state of smartwatch-based ECG research. While accuracy and potential benefit in terms of early detection and accessibility are evident, significant gaps remain in compatibility with existing clinical workflows and robust data governance frameworks that address privacy, security, and regulatory compliance. Overcoming these limitations is crucial for the responsible and effective deployment of wearable ECG technology in HaH programs.

The “Ethical Challenges” with wearable ECGs for HaH highlight the accuracy concerns around potential false positives and negatives and the necessity for further validation across different population groups. The benefits include the possibility of early AF detection, but successful implementation requires clinician involvement and patient education. The sources also raise important points about compatibility, emphasizing the role of clinicians and the need for users to understand how their data are handled. Furthermore, we need to extensively discuss data governance issues, including privacy, security, ownership, and the increasing privatization of research data collected by private companies.

Future research should prioritize several key areas. Firstly, the development of advanced signal processing and noise cancellation algorithms specifically tailored for wearable ECG devices is essential to improve data quality and enhance the accuracy of AI-ECG interpretations. Secondly, further investigation into the clinical validation of multi-lead ECG approximations using wearable devices, particularly for the early detection of critical conditions like myocardial infarction, is warranted in larger and more diverse patient populations. Thirdly, studies focusing on the seamless compatibility of wearable ECG data with electronic health records and the development of user-friendly interfaces for both patients and clinicians are necessary for practical integration into clinical workflows. Finally, robust frameworks for data governance, addressing privacy, security, and regulatory requirements specific to remote cardiac monitoring data, must be established to ensure patient trust and facilitate wider adoption.

Furthermore, exploring the potential of leveraging multimodal data, such as combining ECG signals with accelerometry and other physiological parameters, through advanced AI techniques like reinforcement learning, could unlock new insights into cardiac health and lead to more comprehensive and personalized monitoring strategies. Future AI-ECG research should also focus on analyzing the nuances of model predictions to better understand the underlying electrocardiographic features and continuously improve diagnostic accuracy and reduce false alarms. By strategically addressing these research directions, the field can move closer to realizing the full potential of wearable ECG devices in preventing cardiovascular disease, improving patient outcomes, and enabling effective “Hospital at Home” care.

## Figures and Tables

**Table 1:** Evaluation metrics for k-nearest neighbors (KNN), Decision Tree (DT), Support Vectorial Machine (SVM), Random Forest (RF), Convolutional Neural Network (CNN) [26].

Algorithm	Accuracy%	Precision%	Recall%	F1-Score%
KNN	98%	99%	98%	98%
DT	99%	99%	99%	98%
SVM	92%	59%	87%	68%
RF	95%	95%	96%	95%
LR	77%	77%	78%	77%
CNN	98%	98%	99%	98%

**Table 2:** Classification of arrhythmic images into 8 categories: premature atrial contractions (APC) with 2544 instances, left bundle branch block (LBBB) with 8072 instances, normal sinus rhythm (NOR) with 10832 instances, premature atrial beats (PAB) with 7024 instances, premature ventricular contractions (PVC) with 7130 instances, right bundle branch block (RBBB) with 7256 instances, and ventricular ectopic beat (VEB) with 106 instances [43].

	Precision	Recall	F1-Score
APC	100%	87%	93%
NOR	100%	96%	98%
LBBB	99%	100%	99%
PAB	99%	100%	100%
PVC	92%	99%	95%
RBBB	99%	98%	99%
VEB	100%	100%	100%
Accuracy			98%
Macro Avg	98%	97%	98%
Weighted Avg	98%	98%	98%

**Table 3:** Results from selected publications showing the pros and cons associated with the use of ECG wearable devices.

Reference	Delimitation	Pros	Cons
Choi et al. [51]	Apple Watch Series 4 with the assistance of the attending physician on duty.	It assesses three QCG features: qSTEMI for acute occlusive myocardial infarction (STEMI), qACS for acute coronary syndrome (ACS) including STEMI, non-STEMI, and unstable angina, and qMI for elevated cardiac troponin I.	Each single-lead ECG acquisition takes 30 s, and a total of 4 min and 30 s are required for the smartwatch ECG; however, due to noise or the need for repeated measurements, this process is sometimes extended to 5–10 min. The data are in image format, not digital signals.
Hilbel et al. [6]	FitBit © Sense, Apple © watch series 4, Samsung © Galaxy Watch 4, and Withings © ScanWatch Devices.	ECG devices allow reliable and timely detection of atrial fibrillation and can protect against stroke.	Currently, neither single-channel nor 6-channel ECG are adequate or sufficient for the general detection of myocardial ischemia.
Abu-Alrub, et al. [2]	Apple Watch Series 5^®^, Samsung Galaxy Watch Active 3^®^, and Withings Move ECG^®.^	The use of wearable devices with ECG technology has spread, which allows detecting symptomatic and asymptomatic AF.	The accuracy of algorithms for diagnosing AF varies between smartwatch models, as does the quality of ECG graphs recorded for interpretation by healthcare professionals.
Rjoob et al. [42]	Machine Learning/Deep Learning 2000–2019.	There is significant variability in the different metrics: precision, sensitivity, specificity, and AUC. According to the results of the meta-analysis, ECG analysis using ML and DL showed promising results, especially in the detection of cardiac abnormalities.	ML in general and DL in particular do not perform well when using small datasets. Experts suggest that there is an inverse relationship between ML performance/accuracy and explainability, where higher-performing techniques such as DL are less explainable.
Saadatnejad et al. [44]	The study’s scope was narrowed to evaluating the feasibility of implementing this approach on specific wearable devices, namely Moto 360, Nano Pi Neo Plus 2, and Raspberry Pi Zero.	The model demonstrated exceptional accuracy with arrhythmic diseases such as VEB, LBBB, and PAB achieving prediction metrics above 99% in precision, recall, and F1-score.	The model’s performance heavily relies on the quality and representativeness of the training data. Biased or incomplete data can lead to models that do not perform well across different demographics or clinical situations.
Strik et al. [48]	ECG smartwatch for arrhythmia detection.	Potential use of smartwatch ECGs beyond the diagnosis of tachycardia, in the detection of ischemia, bradycardia, QT prolongation, and abnormalities associated with sudden cardiac death.	Lead I, V1, V3, V6 using different smartwatch placements.
Samol et al. [50]	Single-lead ECG recordings including Einthoven and Wilson leads.	lead I = Einthoven lead I, lead II = Einthoven lead II, lead III = Einthoven lead III, Wr = pseudo-unipolar recording corresponding to standard lead V1, Wm = unipolar recording corresponding to standard lead V4, Wl = pseudo-unipolar recording corresponding to standard lead V6.	Lead I, II, III, V1, V4, V6 through different placements of the smartwatch.

**Table 4:** Results from the previously selected publications show the detection capabilities associated with each of the selected wearable devices.

Device	Single-Lead Type I ECG	6-Lead ECG Using a Third Electrode	Sensitivity and Specificity for Diagnosing AF	Detection of ST/T Wave Abnormalities	Different Placements of the Smartwatch	References
FitBit Sense ^1^	✅		68%/98%			[6]
Apple Watch Series 4 ^2^	✅		99%/92%	I, II, II, V1, V4, V6	✅	[48,50]
Apple Watch Series 5 ^2^	✅		99%/92%	I, II, II, V1, V4, V6	✅	[2]
Samsung Galaxy Watch Active 3 ^3^	✅		96%/98%			[2]
Samsung Galaxy 4 ^3^	✅		96%/98%			[2]
Withings Smart Body Scale ^4^	✅	✅		I, II, III		[6]
Withings Move ECG ^4^	✅		94%/94%			[2]
Kardia Alive Cor 6L ^5^	✅	✅		I, II, III		[6]
Moto 360 ^6^, Nano Pi Neo Plus 2 ^7^, and Raspberry Pi Zero ^8^	✅	✅	99%/99%	I		[44]

Notes: ^1^ Fitbit LLC (a subsidiary of Google LLC), Mountain View, CA, USA; ^2^ Apple Inc, Cupertino, CA, USA; ^3^ Samsung, Seoul, South Korea; ^4^ Withings, Issy-les-Moulineaux, France; ^5^ Alive Cor, Inc., Mountain View, CA, USA; ^6^ Motorola Mobility LLC, Chicago, IL, USA; ^7^ FriendlyElec, Shenzhen, Guangdong, China; ^8^ Raspberry Pi Foundation, Cambridge, Cambridgeshire, UK.

**Table 5:** Results from the previously selected publications show how the reviewed literature aligns with the ABCD framework.

Reference	Accuracy	Benefit	Compatibility	Data
Choi et al. [51]	✅ Explores accuracy of smartwatch ECG with AI for detecting acute coronary syndrome (ACS) compared to 12-lead ECG.	✅ Potential for early detection of ACS using smartwatches with AI, leading to timely intervention.	✅ Explores the use of smartwatch ECG, implying potential for personal and home use.	✅ Involves ECG data and application of AI algorithms for detecting ACS.
Sarma et al. [54]	✅ Machine Learning (ML) methods can improve the interpretation of diagnostic tests such as ECGs.	✅ AI technology may enhance routine clinical care. ML-based risk stratification and prognostication may help optimize triaging.	✅ Focuses on applications within the Cardiac Intensive Care Unit (CICU).	✅ Leverages electronic health record (EHR) data.
Bayoumy et al. [3]	✅ Discusses device accuracy and limitations.	✅ Evaluates clinical utility.	✅ Considers integration into workflow.	✅ Addresses data privacy concerns.
Abu-Alrub et al. [2]	✅ Compares smartwatch AF detection accuracy.	✅ Demonstrates clinical relevance for AF detection.	❎ No direct mention of workflow integration.	❎ Limited discussion on data governance.
Strik et al. [48]	✅ Examines positioning for better ECG accuracy.	✅ Suggests improvement in ST/T wave detection.	✅ Discusses alternative recording positions.	❎ No mention of data privacy.
Samol et al. [50]	✅ Analyzes smartwatch-derived multi-lead ECGs.	✅ Explores smartwatch ECG feasibility for MI detection.	✅ Investigates usability for emergency diagnosis.	❎ Limited focus on data security.
Predel et al. [55]	✅ Discusses potential for false positives and false negatives in AF detection by smartwatches. Requires further research on algorithm accuracy for different populations.	✅ Potential for early detection and monitoring of atrial fibrillation, potentially improving the doctor–patient relationship with patient education and clinician involvement.	✅ Emphasizes the need for clinician leadership in implementation and patient education about early detection and risks. Raises concerns about users not understanding data storage and usage.	✅ Highlights concerns about data storage, ownership, potential misuse by private companies, and the need for data protection laws—Points out the privatization of research data.

## Data Availability

Not applicable.

## References

[1] Hayes S., Salzberg C., McCharty D. High-Need, High-Cost Patients: Who Are They and How Do They Use Health Care? 29 August 2016. https://www.commonwealthfund.org/publications/issue-briefs/2016/aug/high-need-high-cost-patients-who-are-they-and-how-do-they-use.

[2] Abu-Alrub S., Strik M., Ramírez F. (2022). Smartwatch Electrocardiograms for Automated and Manual Diagnosis of Atrial Fibrillation: A Comparative Analysis of Three Models. Front. Cardiovasc. Med..

[3] Bayoumy K., Gaber M., Elshafeey A., Mhaimeed O., Dineen E.H., Marvel F.A., Martin S.S., Muse E.D., Turakhia M.P., Tarakji K.G. (2021). Smart wearable devices in cardiovascular care: Where we are and how to move forward. Nat. Rev. Cardiol..

[4] Caillol T., Strik M., Ramírez F., Abu-Alrub S., Marchand H., Buliard S. (2021). Accuracy of a smartwatch-derived ECG for diagnosing bradyarrhythmias, tachyarrhythmias, and cardiac ischemia. Circ. Arrhythm. Electrophysiol..

[5] Strik M., Caillol T., Ramirez F., Abu-Alrub S., Marchand H., Welte N. (2020). Validating QT-interval measurement using the apple watch ECG to enable remote monitoring during the COVID-19 pandemic. Circulation.

[6] Hilbel T., Frey N. (2023). A Review of current ECG consumer electronics (pros and cons). J. Electrocardiol..

[7] Ornato J., Riddell J., Carley S., Mackway-Jones K., Peberdy M. (2022). 80-lead body map detects acute ST elevation MI missed by standard 12-lead. J. Am. Coll. Cardiol..

[8] McClelland A., Owens C., Menown I., Lown M., Adgey A. (2023). Comparison of the 80-lead body surface map to physician and to 12-lead electrocardiogram in detection of acute myocardial infarction. Am. J. Cardiol..

[9] Owens C., McClelland A., Walsh S., Smith B., Tomlin A., Riddell J. (2004). Prehospital 80-LAD mapping: Does it add significantly to the diagnosis of acute coronary syndromes?. J. Electrocardiol..

[10] Escudero P., Cabanas A., Dotor-Castilla M. (2023). Are ActivityWrist-Worn Devices Accurate for Determining Heart Rate during Intense Exercise?. Bioengineering.

[11] Thompson W. (2019). Worldwide survey of fitness trends for 2020. Acsm’s Health Fit. J..

[12] Thompson W. (2021). Worldwide Survey of Fitness Trends for 2021. Acsm’s Health Fit. J..

[13] Thompson W. (2022). Worldwide Survey of Fitness Trends for 2022. Acsm’s Health Fit. J..

[14] Shei R., Holder I., Oumsang A., Paris B., Paris H. (2022). Wearable activity trackers-advanced technology or advanced marketing?. Eur. J. Appl. Physiol..

[15] Allen J. (2007). Photoplethysmography and its application in clinical physiological measurement. Physiol. Meas..

[16] Alian A., Shelley K. (2014). Photoplethysmography. Best Pract. Res. Clin. Anaesthesiol..

[17] Lee I., Park N., Lee H., Hwang C., Kim J., Park S. (2021). Systematic Review on Human Skin-Compatible Wearable Photoplethysmography sensors. Appl. Sci..

[18] Zakeri I., Adolph A., Puyau M., Vohra F., Butte N. (2008). Application of cross-sectional time series modeling for the prediction of energy expenditure from heart rate and accelerometry. J. Appl. Physiol..

[19] Chowdhury E., Western M., Nightingale T., Peacock O., Thompson D. (2017). Assessment of laboratory and daily energy expenditure estimates from consumer multisensor physical acitivity monitors. PLoS ONE.

[20] Perez M., Mahaffey K., Hedlin H., Rumsfeld J., Garcia A., Ferris T. (2019). Large-scale assessment of a smartwatch to identify atrial fibrillation. N. Engl. J. Med..

[21] Rajakariar K., Koshy A., Sajeev J., Nair S., Roberts L., Teh A. (2020). Accuracy of a smartwatch based single-lead electrocardiogram device in detection of atrial fibrillation. Heart Br. Card. Soc..

[22] Mannhart D., Hennings E., Lischer M. (2022). Clinical Validation of Automated Corrected QT-Interval Measurements from a Single Lead Electrocardiogram Using a Novel Smartwatch. Front. Cardiovasc. Med..

[23] Spaccarotella C., Migliarino S., Mongiardo A., Sabatino J., Santarpia G., de Rosa S. (2021). Measurement of the QT interval using the apple watch. Sci. Rep..

[24] Garabelli P., Stavrakis S., Albert M., Koomson E., Parwani P., Chohan J. (2016). Comparison of QT interval readings in normal sinus rhythm between a smartphone heart monitor and a 12-lead ECG for healthy volunteers and inpatients receiving sotalol or dofetilide. J. Cardiovasc. Electrophysiol..

[25] Wan E., Ghanbari H., Akoum N., Itzhak Attia Z., Asirvatham S., Chung E. (2021). HRS white paper on clinical utilization of digital health technology. Cardiovasc. Digit. Health J..

[26] Salama D., Gamal A., Mohamed Y. DeepECG: Biulding an Efficient Framework for Automatic Arrythmia classification model. Proceedings of the 2nd International Mobile, Intelligent, and Ubiquitous Computing Conference (MIUCC).

[27] Zepeda-Echavarria A., van de Leur R., van Sleuwen M. (2023). Electrocardiogram Devices for Home Use: Technological and Clinical Scoping Review. JMIR Cardio.

[28] Dahiya E.S., Kalra A.M., Lowe A., Anand G. (2024). Wearable Technology for Monitoring Electrocardiograms (ECGs) in Adults: A Scoping Review. Sensors.

[29] Lu L., Zhang J., Xie Y. (2020). Wearable Health Devices in Health Care: Narrative Systematic Review. JMIR Mhealth Uhealth.

[30] Naksuk N., Lazar S., Peeraphatdit T. (2020). Cardiac safety of off-label COVID-19 drug therapy: A review and proposed monitoring protocol. Eur. Heart J. Acute Cardiovasc..

[31] Ferguson C., Hickman L., Turkmani S. (2021). Wearables only work on patients that wear them: Barriers and facilitators to the adoption of wearable cardiac monitoring technologies. Cardiovasc. Digit. Health J..

[32] Hindricks G., Potpara T., Dagres N., Arbelo E., Bax J., Blomström-Lundqvist C. (2021). The Task Force for the diagnosis and management of atrial fibrillation of the European Society of Cardiology (ESC). Eur. Heart J..

[33] Meo M., Zarzoso V., Latcu D., Saoudi N. (2013). Spatial variability of the 12-lead surface ECG as a tool for noninvasive prediction of catheter ablation outcome in persistent atrial fibrillation. IEEE Trans. Biomed. Eng..

[34] Samol A., Bischof K., Luani B., Pascut D., Wiemer M., Kaese S. (2019). Recording of Bipolar Multichannel ECGs by a Smartwatch: Modern ECG Diagnostic 100 Years after Einthoven. Sensors.

[35] Han C., Song Y., Lim H.-S., Tae Y., Jang J.-H. (2021). Automated Detection of Acute Myocardial Infarction Using Asynchronous Electrocardiogram Signals—Preview of Implementing Artificial Intelligence with Multichannel Electrocardiographs Obtained From Smartwatches: Retrospective Study. J. Med. Internet Res..

[36] Silva M., Goncalves H., Almeida R. (2024). Cardiovascular responses as predictors of mortality in children with acute brain injury. Pediatr. Res..

[37] Burma J., Griffits J., Lapointe A. (2024). Heart Rate Variability and Pulse Rate Variability: Do Anatomical Location and Sampling Rate Matter?. Sensors.

[38] Ardeti V., Kolluru V., Varguese G., Patjoshi R. (2023). An overview on state-of-the-art electrocardiogram signal processing meth-ods: Traditional to AI-based approaches. Expert Syst. Appl..

[39] Baseer K., Sivakumar k., Veeraiah D. (2024). Healthcare diagnostics with an adaptive deep learning model integrated with the Internet of medical Things (IoMT) for predicting heart disease. Biomed. Signal Process. Control.

[40] Prajitha C., Sridhar K., Baskar S. (2022). ECG diagnosis for arrhythmia detection with a cloud-based service and a wearable sensor network in a smart city environment. Front. Sustain. Cities.

[41] Li W., Ming-Tang Y., Yu K. (2022). SLC-GAN: An automated myocardial infarction detection model based on generative adversarial networks and convolutional neural networks with single-lead electrocardiogram synthesis. Inf. Sci..

[42] Rjoob K., Bond R., Finlay D., McGilligan V., Leslie S. (2022). Machine learning and the electrocardiogram over two decades: Time series and meta-analysis of the algorithms, evaluation metrics and applications. Artif. Intell. Med..

[43] Sanjay T., Shashidhar R. ECG based Heart Disease Classification and Validation using 2D CNN. Proceedings of the 5th International Conference on Contemporary Computing and Informatics (IC3I).

[44] Saadatnejad S., Oveisi M. (2020). LSTM-Based ECG Classification for Continuous Monitoring on Personal Wearable Devices. IEEE J. Biomed. Health Inform..

[45] Tang K., Ma S., Sun X., Guo D. (2024). Optimizing machine learning for enhanced automated ECG analysis in cardiovascular healthcare. Egypt. Inform. J..

[46] Rahman M.S., Karmarkar C., Islam S.M.S. (2024). Application of Federated Learning in Cardiology: Key Challenges and Potential Solutions. Mayo Clin. Proc. Digit. Health.

[47] Yu-Lan L., Chin-Sheng L. (2022). A Deep Learning Algorithm for Detecting Acute Pericarditis by Electrocardiogram. J. Pers. Med..

[48] Strik M., Ploux M.S., Weigel D. (2023). The use of smartwatch electrocardiogram beyond arrhythmia detection. Trends Cardiovasc. Med..

[49] Nasarre M., Strik M., Ramirez D., Buliard S., Marchand H., Abu-Alrub S. (2021). Using a smartwatch electrocardiogram to detect abnormalities associated with sudden cardiac arrest in young adults. EP Europace.

[50] Samol A., Bsichof K., Luiani B., Pascut D., Wiemer M., Kaese S. (2019). Single-Lead ECG Recordings Including Einthoven and Wilson Leads by a Smartwatch: A New Era of Patient Directed Early ECG Differential Diagnosis of Cardiac Diseases?. Sensors.

[51] Choi J., Kim J., Spaccarotella C., Esposito G., Oh I.-Y., Cho Y., Indolfi C. (2025). Smartwatch ECG and artificial intelligence in detecting acute coronary syndrome compared to traditional 12-lead ECG. IJC Heart Vasc..

[52] Maille B., Wilkin M., Million M., Rességuier N. (2021). Smartwatch Electrocardiogram and Artificial Intelligence for Assessing Cardiac-Rhythm Safety of Drug Therapy in the COVID-19 Pandemic. The QT-logs study. Int. J. Cardiol..

[53] Roden D., Harrington R., Poppas A., Russo A. (2020). Considerations for drug interactions on QTc interval in exploratory COVID-19 treatment. J. Am. Coll. Cardiol..

[54] Sarma D., Rali A.S., Jentzer J.C. (2025). Key Concepts in Machine Learning and Clinical Applications in the Cardiac Intensive Care Unit. Curr. Cardiol. Rep..

[55] Predel C., Steger F. (2020). Ethical Challenges with Smartwatch-Based Screening for Atrial Fibrillation: Putting Users at Risk for Marketing Purposes?. Front. Cardiovasc. Med..

